# Characterization of the complete chloroplast genome sequence of *Lycium qingshuiheense* (Solanaceae)

**DOI:** 10.1080/23802359.2024.2341113

**Published:** 2024-04-15

**Authors:** Bo Zhang, Wangsuo Liu, Xueli Ding, Wei Zhang, Jinzhong Zhu, Xuejun Wang

**Affiliations:** a Ningxia Technical College of Wine and Desertification Prevention, Yinchuan, China; b Department of Chemical and Environmental Engineering, Hetao College, Bayannur, China; c Qixin Wolfberry Seedling Professional Cooperative of Zhongning County, Zhongwei, China

**Keywords:** Complete chloroplast genome, *Lycium qingshuiheense*, phylogenetic analysis, Solanaceae

## Abstract

*Lycium qingshuiheense* is a typical drought and salt-alkali-tolerant plant, which has been added to the new species of *Lycium* in recent years. Here, we first sequenced the complete chloroplast genome of *L. qingshuiheense* to investigate its evolutionary relationship within the family Solanaceae. Results suggested that the circular complete chloroplast genome of *L. qingshuiheense* was 154,945 bp in length, including a large single-copy (LSC) of 85,930 bp, a small single-copy (SSC) of 18,203 bp, and two inverted repeats (IRs) of 25,406 bp. The GC content accounts for 37.90% and annotated 131 genes, including 86 protein-coding genes, eight rRNA genes, and 37 tRNA genes. A neighbor-joining phylogenetic tree revealed that *L. qingshuiheense* was a sister species to *L. ruthenicum.* Our study provides a new insight into the systematic evolution of *Lycium* in the Solanaceae family.

## 1. Introduction

**Figure 1.**
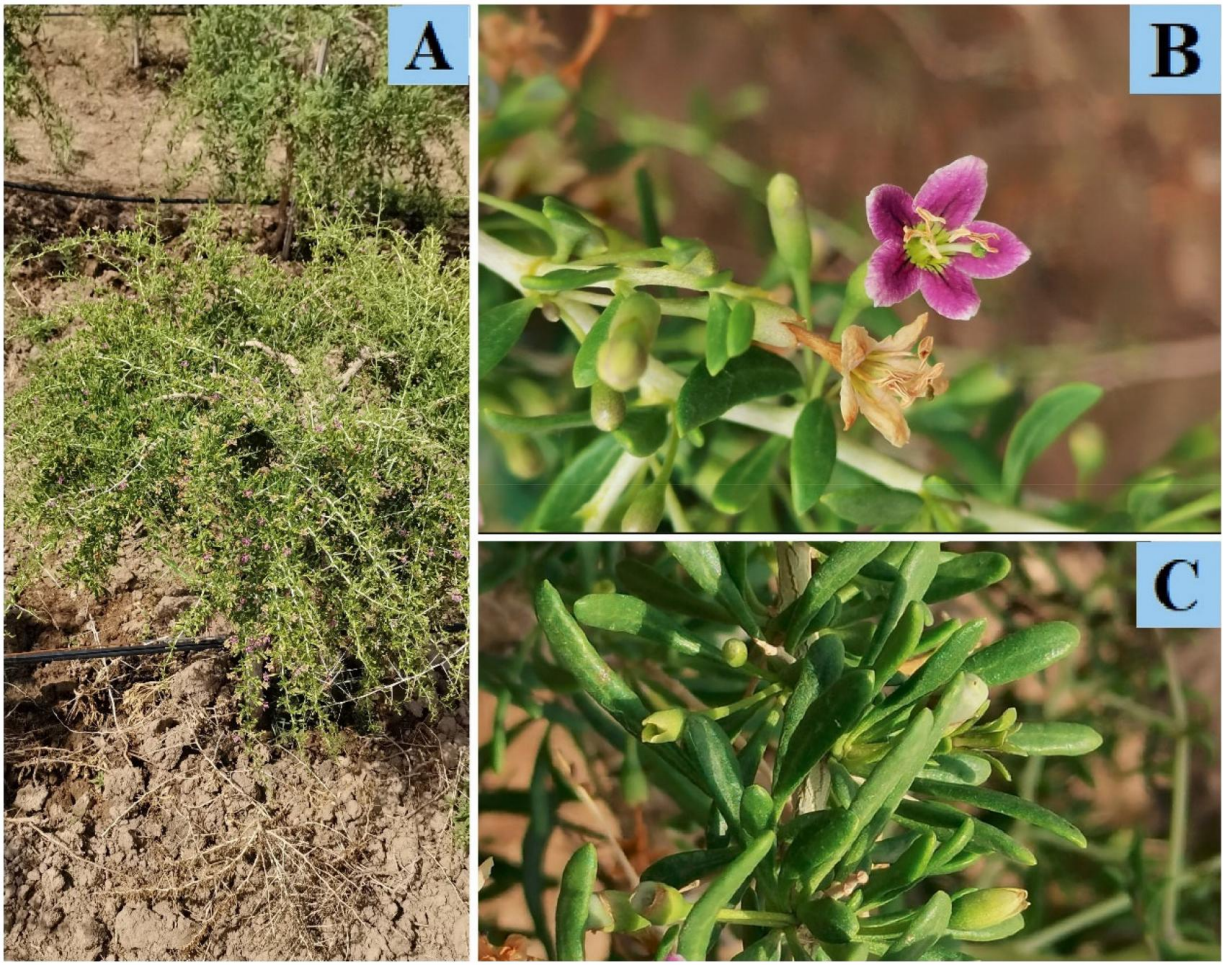
Morphological characteristics of *L. qingshuiheense* (A: the whole plant; B: flower; C: leaf) (these physical photos were taken by the corresponding author, Associate Professor Liu Wangsuo, without publication or infringement).

*Lycium qingshuiheense* X. L. Jiang & J. N. Li in 2011, a new species of *Lycium* in the family Solanaceae, is sparsely distributed in Ningxia and Gansu provinces of northwest China (Li et al. 2011; Cui et al. 2013). The berry of this species appears compressed-globose and darkly red-brown (Li et al. 2011), rich in a variety of vitamins and trace elements (Liu et al. 2016), as well as showing excellent drought tolerance and saline-alkali tolerance. The leaves of *L. qingshuiheense* are thick and the inner side of the corolla is purple-pink, and calyx with irregularly 2–4 lobed (Figure 1). It has been identified as an important resource of traditional Chinese medicine from the fourth national general investigation of natural resources of Chinese materia medica (Cui et al. 2013). The gene fragments (*accD*, *matk*, *rps16-trnQ*, *psbA-trnH*, etc.) in plastids are commonly used to mark phylogenetic status and taxonomic attributes of species (Mishra et al. 2015; Van Do et al. 2021). To facilitate its phylogenetic research and contribute to development and utilization, it is urgent to take appropriate measures to protect this rare medicinal resource. In the present study, we determined the complete chloroplast genome of *L. qingshuiheense* to analyze its phylogenetic position, as well as to provide useful information for further studies.

## 2. Materials and methods

Fresh leaves of *L. qingshuiheense* were sampled from Qingshui River Basin, Zhongning County, Ningxia Hui Autonomous Region, China (37°28′29.94 N, 105°32′29.92 E, alt. 1150.7 m). A voucher specimen (2023LQSH001) was deposited at the Plant and Plant Physiology Laboratory, Ningxia Technical College of Wine and Desertification Prevention (http://www.nxfszs.cn/, Wangsuo Liu, email: liuwangsuo@sina.com). Total genomic DNA was extracted according to the modified CTAB method of Stefanova et al. (2013), and stored in the −80 °C refrigerator in the laboratory. The genome sequencing was conducted by Illumina Hiseq 2500 at Biomarker Technologies Corporation (Beijing, China). Filtered the low-quality sequences, and high-quality sequences were assembled by the assembler SPAdes3.11.0 (Nurk et al. 2013). The annotation was performed by Plann (Huang and Cronk 2015). Then, the complete chloroplast genome map was drawn by CPGView program (http://www.1kmpg.cn/cpgview/) (Liu et al. 2023). Comparison of the junction of LSC, SSC, and IR regions of *L. qingshuiheense* was performed using R (version 4.2.0) to link to the IRscope website (https://irscope.shinyapps.io/irapp/) (Amiryousefi et al. 2018). The chloroplast genomes of 26 *Lycium* in Solanaceae and two outgroups were downloaded from the NCBI database. The downloaded chloroplast genome sequence was aligned with *L. qingshuiheense* using MAFFT-7.037 (Katoh and Standley 2013). A neighbor-joining phylogenetic tree was constructed by MEGA-X (Kumar et al. 2018) based on 28 species. The sequence of *L. qingshuiheense* complete chloroplast genome has been submitted to GenBank (accession number OR551484).

## 3. Results

**Figure 2.**
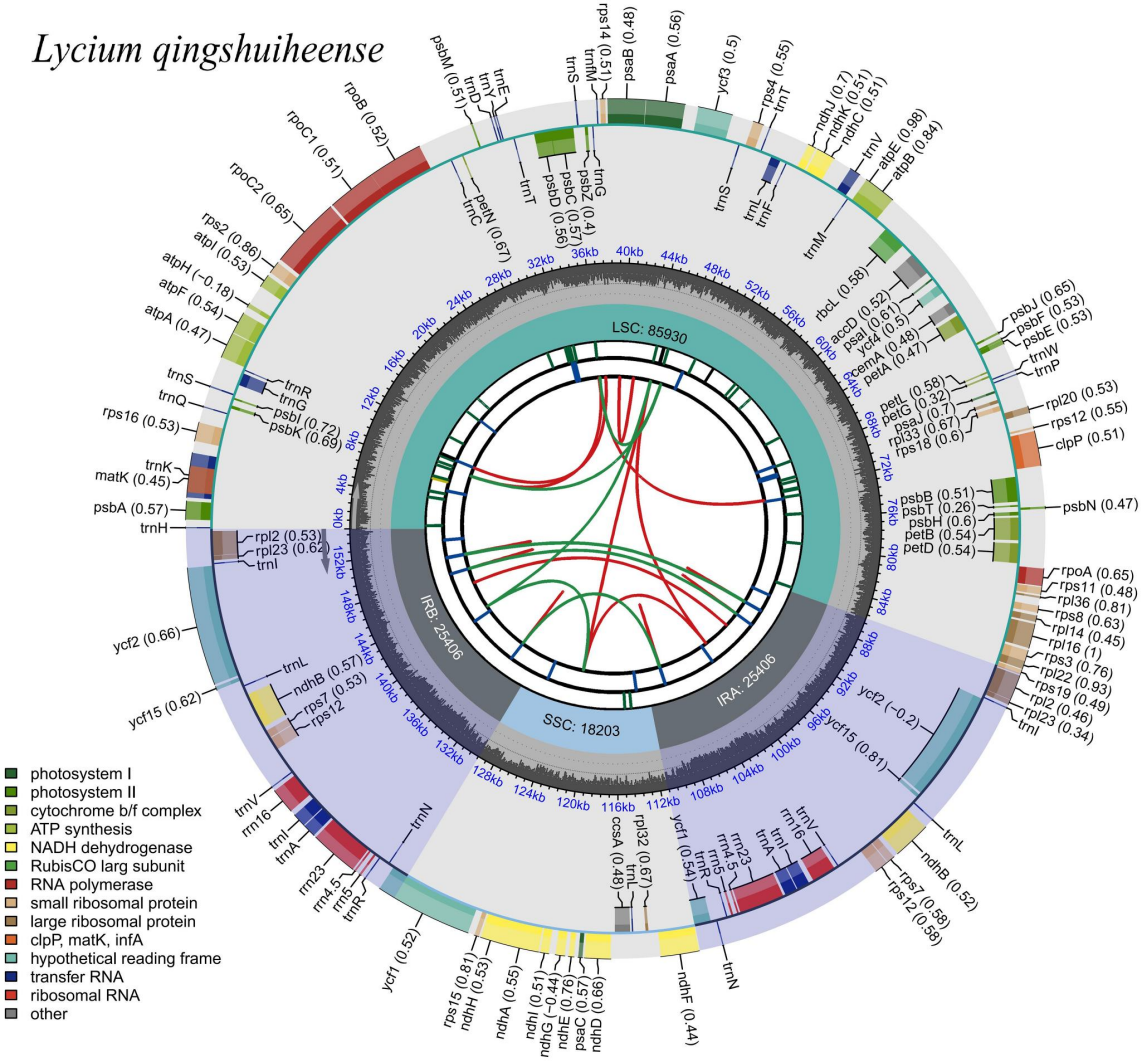
Gene map of *L. qingshuiheense* chloroplast genome. Genes shown inside the circle indicate that the direction of transcription is clockwise, while those shown outside are counterclockwise. Different groups of functional genes are indicated in different colors. The GC content is shown in the dashed area in the inner circle.

**Figure 3.**
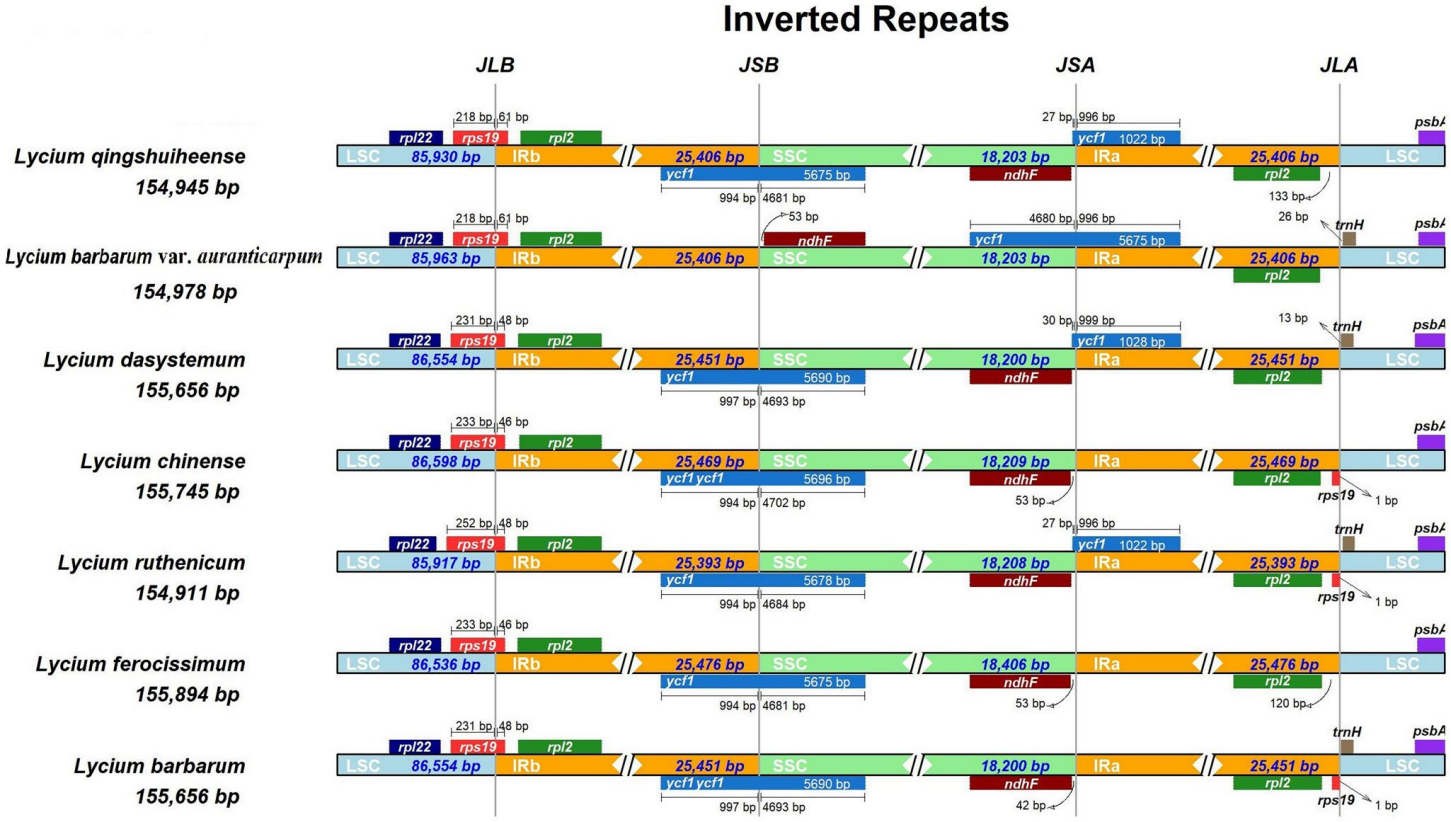
Comparison of borders of LSC, IR, and SSC regions in the chloroplast genomes between *L. qingshuiheense*, *L. barbarum* var. *auranticarpum* (OP846044), *L. dasystemum* (NC067978), *L. chinense* (MN102357) (He et al. 2020), *L. ruthenicum* (MK994503) (Wang et al. 2019), *L. ferocissimum* (MN866909) (Li et al. 2020), and *L. barbarum* (MH032560) (Jia et al. 2018). The junction line between LSC and IRb is abbreviated as JLB; the junction line between SSC and IRb is abbreviated as JSB; the junction line between SSC and IRa is abbreviated as JSA; the junction line between LSC and IRa is abbreviated as JLA.

**Figure 4.**
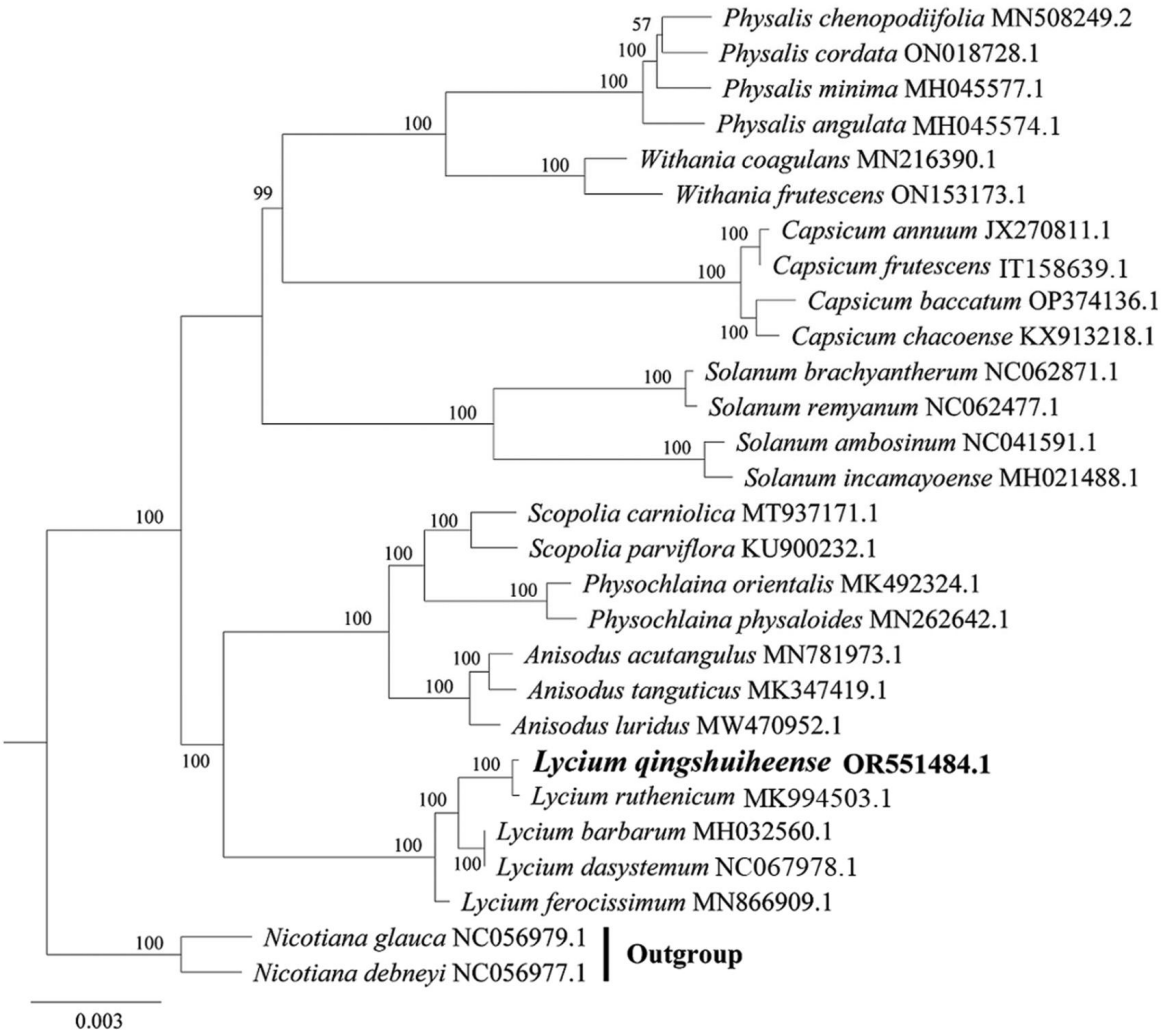
Neighbor-joining phylogenetic tree based on 26 complete chloroplast genomes and two outgroups. Among this species, *Physalis chenopodifolia* (MN508249) (Zamora-Tavares et al. 2020), *P. minima* (MH045577) (Feng et al. 2020), *P. angulata* (MH045574) (Feng et al. 2020), *Capsicum annuum* (NC018552) (Bie et al. 2020), *C. frutescens* (IT158639) (Shim et al. 2016), *Physochlaina physaloides* (MN262642) (Tong et al. 2019), *Anisodus acutangulus* (MN781973) (Tian et al. 2020), *Lycium barbarum* (MH032560) (Jia et al. 2018), *L. ruthenicum* (MK994503) (Wang et al. 2019), and *L. ferocissimum* (MN866909) (Li et al. 2020) have been published. The number on each node represents bootstrap values.

The *L. qingshuiheense* chloroplast genome is 154,945 bp in length, including two inverted repeat (IR) regions (25,406 bp) that are separated by a small single-copy region (18,203 bp) and a large single-copy region (85,930 bp). GC content of the chloroplast genome of *L. qingshuiheense* is 37.90%, with 43.2%, 32.3%, and 36.0% in the IR, SSC, and LSC regions, respectively. The chloroplast genome contains 131 single genes, including 86 protein-coding genes (CDS), eight rRNA, and 37 tRNA genes (Figure 2). Among these genes, the majority are single copy, whereas eight CDS (*rps12*, *rps7*, *rp123*, *rp12*, *ycf1*, *ycf2*, *ycf15*, and *ndhB*), four rRNAs (*rrn-16S*, *rrn-23S*, *rrn4.5S*, and *rrn5S*), and seven tRNAs (*trnA-UGC*, *trnI-CAU, trnI-GAU*, *trnL-CAA*, *trnN-GUU*, *trnR-ACG*, and *trnV-GAC*) occur as double copies. The comparison of LSC, SSC, and IR region between *L. qingshuiheense* and its relatives showed that the expansion and contraction of IRa/SSC and Irb/SSC boundaries were usually located in the coding regions of *ycf1* and *rps19* genes. The IR/LSC and IR/SSC boundaries of *L. qingshuiheense* were similar to the related species, such as *L. dasystemum* (NC067978) and *L. ferocissimum* (MN866909), but in addition to *L. barbarum* var. *auranticarpum* (OP846044) (Figure 3). The phylogenetic tree indicated that *L. qingshuiheense* was a sister species to *L. ruthenicum*, and also suggested that *Lycium* was closely related to *Anisodus*, *Physochlaine*, and *Scopolia* (Figure 4).

## 4. Discussion

Previous studies have reported that the complete chloroplast genome of *L. barbarum*, *L. chinense*, and *L. ruthenicum* were 155,656, 155,745, and 154,869 bp in length, respectively, each containing 133 genes (Cui et al. 2019). In our study, the length of *L. qingshuiheense* was 154,945 bp, which was close to that of *L. ruthenicum*, but contains 131 genes. Three regions with high variation, *atpH-atpI*, *accD-ycf4*, and *ndhF-trnL*, were found in *L. barbarum*, *L. chinense*, and *L. ruthenicum*, and these regions can be used to mark and identify systematic variation among these species (Cui et al. 2019), future studies of our study will focus on the analysis and comparison of high variable regions between *L. qingshuiheense* and other *Lycium* to explore their phylogenetic differences. Boundary expansion and contraction are considered to be the key reasons for the occurrence of high-variation regions (Konhar et al. 2019). The IRa/SSC and Irb/SSC boundaries are generally positioned in the coding region of *ycf1* and *rps19* genes, which is easy to cause the duplication of these genes, resulting in pseudogenes with different sizes of these boundaries, it is similar to the results of Cui et al. (2019). In general, the contractive and expansive boundaries of *L. qingshuiheense* were similar to those of *L. ruthenicum*, and the source of their migration was likely closer. It indicates the similarity and uniqueness of chloroplast genomes between *L. qingshuiheense* and its closest relatives in phylogenetic evolution.

## 5. Conclusions

In this study, the chloroplast genome of *L. qingshuiheense* was first sequenced, and the annotated circular structure map was obtained. The data of *L. qingshuiheense* are of great reference value for the evaluation of genetic variation, phylogeny, classification recognition, and DNA markers of this species. In addition, this study is also a supplement and improvement of the evolutionary data of *Lycium* species, which lays a foundation for future research.

## Supplementary Material

Supplemental Material

Supplemental Material

## Data Availability

The data that support the findings of this study are openly available in the NCBI database at https://www.ncbi.nlm.nih.gov/, reference number OR551484. The associated BioProject, SRA, and Biosample numbers are PRJNA1016474, SRR26057891, and SAMN37386557, respectively.
