# Supplementary figures and images for: Characterization of the complete chloroplast genome sequence of Lycium qingshuiheense (Solanaceae)
Source: Mitochondrial DNA B Resour. 2024 Apr 15;9(4):517–21. doi: 10.1080/23802359.2024.2341113 (PMC11020591; doi:10.1080/23802359.2024.2341113)

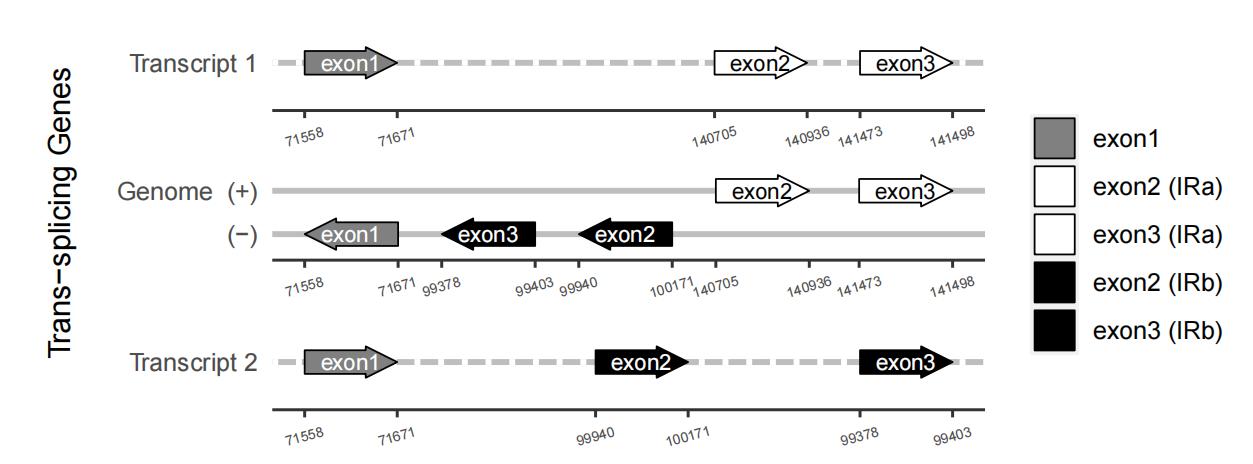

Supplement: Supplemental Material [file TMDN_A_2341113_SM7916.jpg]

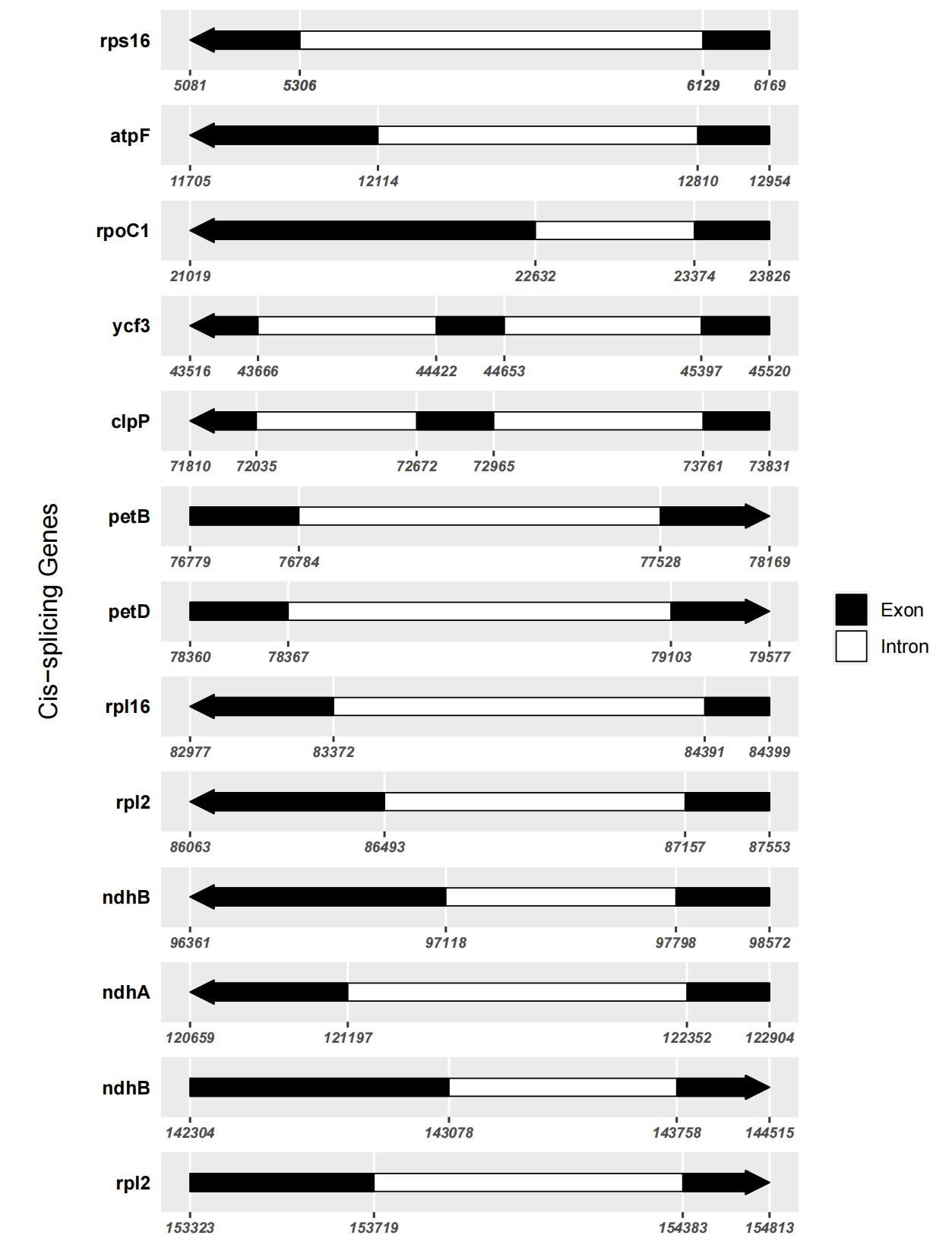

Supplement: Supplemental Material [file TMDN_A_2341113_SM7908.jpg]
